# Why does subordinates’ negative workplace gossip lead to supervisor undermining? A moderated mediation model

**DOI:** 10.3389/fpsyg.2022.981539

**Published:** 2022-09-29

**Authors:** Hao Zeng, Lijing Zhao, Jinsheng Li

**Affiliations:** ^1^School of Economic and Management, Jiangxi Science and Technology Normal University, Nanchang, China; ^2^School of Management, Hainan University, Haikou, China; ^3^Business School, Guilin University of Technology, Guilin, China

**Keywords:** negative workforce gossip, emotional exhaustion, undermining, mindfulness, conservation of resources theory

## Abstract

**Objectives:**

Previous studies on negative workplace gossip have neglected the role of gossip targets of supervisors. The purpose of this paper is to deepen our understanding of how subordinates’ negative workplace gossip affects supervisors’ work-related behaviors. Drawing upon conservation of resource theory, the authors propose that subordinates’ negative gossip leads to supervisor emotional exhaustion. In turn, such emotional exhaustion provokes supervisors to exhibit undermining toward their subordinates. Additionally, the authors propose that a trait factor, namely, supervisor mindfulness, mitigates the relationship between such negative workplace gossip and supervisors’ emotional exhaustion.

**Method:**

Data were collected from employees (e.g., subordinates) and their immediate supervisors in 35 organizations located in Jiangsu and Anhui Provinces in China. The data were obtained at three time points, each time interval was 2 weeks, and finally, 362 valid data points were obtained.

**Results:**

The following findings were obtained: (1) perceived subordinates’ negative gossip has a significant positive effect on supervisor undermining; (2) supervisor emotional exhaustion mediates the relationship between perceived subordinates’ negative gossip and supervisor undermining; and (3) supervisor mindfulness moderates the relationship between perceived subordinates’ negative gossip and supervisor emotional exhaustion and moderates the mediating effect of supervisor emotional exhaustion on the relationship between perceived subordinates’ negative gossip and supervisor undermining.

**Conclusion:**

Using multisource data and a moderated mediation model, we found that subordinates’ negative workplace gossip predicts supervisor undermining through supervisor emotional exhaustion. We also discovered that supervisor mindfulness can buffer the positive relationship between perceived subordinates’ negative workplace gossip and supervisor undermining. These findings have important implications for the literature on negative gossip in the workforce, especially the impact of subordinates’ negative workplace gossip on supervisors’ responses.

## Introduction

Negative workplace gossip is a common negative organizational behavior that involves negative personal information that employees perceive to be maliciously disseminated or commented on by others in the organization (Chandra and Robinson, 2009). A negative gossip episode involves three parties, including the gossiper, the target, and the recipients of the gossip (Zhou et al., 2020; Dores Cruz et al., 2021). Of the gossipers and gossip recipients’ perspectives (Tassiello et al., 2018; Guo et al., 2021), research on negative workplace gossip from the perspective of the gossip target has received increasing attention (Martinescu et al., 2021; Tan et al., 2021; Cheng et al., 2022). Studies have shown that once an employee perceives that he or she has become the target of negative workplace gossip, his or her emotions, cognitions and behaviors will have a series of negative consequences (Baumeister et al., 2004; Beersma and Van Kleef, 2012), such as physical and mental harm (Wu et al., 2016; Naeem et al., 2019), psychological stress (Demerouti et al., 2001; Zhou et al., 2019), job satisfaction decline, decreased proactive, and innovative behavior (Wu et al., 2015; Tian et al., 2019), and increased deviation behaviors (Michelson and Mouly, 2000; Greengard, 2001), and will ultimately damage organizational climate, organizational performance, and other organizational variables (Akande and Odewale, 1994; Baker and Jones, 1996). Previous studies have mostly investigated employees as gossip targets (e.g., Kong, 2018; Tian et al., 2019; Ye et al., 2019; Yao et al., 2020), and we have a limited understanding of the research on supervisors as gossip targets. In fact, the phenomenon of supervisors becoming the target of gossip is widespread and deserves extensive attention from organizational researchers. The reasons are as follows. Employees with low formal status in the organization pay more attention to employees with higher status in the organization (Tebbutt and Marchington, 1997; McAndrew et al., 2007). Moreover, the gossiper usually spreads negative gossip when the gossip target is absent (Foster and Rosnow, 2006; Dores Cruz et al., 2021), and negative gossiping about managers increases when employees notice the frequent absence of their managers (Ellwardt et al., 2012a). Supervisors’ status in the organization and their engagement with multiple subordinates make it difficult to ensure their full presence, subsequently making them soft targets for subordinates’ gossip at work (Naeem et al., 2019). Furthermore, negative workforce gossip directed at supervisors is a strategic tool for subordinates to align with each other (Tebbutt and Marchington, 1997; Brady et al., 2017). Thus, given that supervisors are potential targets of negative workplace gossip; this study focuses on how negative workplace gossip affects the emotional and behavioral responses of the gossip target (supervisors). This research will help organizational leaders further understand the harm of negative workplace gossip and provide inspiration for mitigating the negative impact of negative workplace gossip, thereby breaking a vicious circle as follows: subordinates’ negative workplace gossip—supervisors’ negative emotions—negative behavior toward subordinates (Naeem et al., 2019)—more perceived negative workplace gossip from subordinates.

Conservation of resources theory points out that individuals have the tendency to acquire, maintain, and protect work resources that are beneficial to them and avoid the loss of these resources (Hobfoll, 1989, 1998). When individual resources (such as psychological, emotional, intellectual, conditional, and other resources) are sufficient, resources are further invested to obtain more resources, and when individual resources are lost and cannot be recovered within a period of time, resource input is reduced (Hobfoll, 2001). As mentioned above, negative workforce gossip as a negative event predicts a suboptimal work environment, relationship dysfunction, and reduced interpersonal trust (Ellwardt et al., 2012a,b); threatens the development of valuable social relationships at work; and can be regarded as a source of stress in interpersonal relationships (Zhou et al., 2019). According to conservation of resources theory, we believe that perceiving subordinates’ negative workforce gossip leads to supervisors losing psychological resources or finding themselves in a state of emotional exhaustion (Liu et al., 2019), with the result that they devote effort to take active defensive measures toward undermining subordinates by deterring them, restoring psychological resources, and preserving reputational, work-related resources that are beneficial to their own (Naeem et al., 2019; Cheng et al., 2022; Yao et al., 2020). We also paid special attention to the situational characteristics of Chinese workplaces. On the one hand, the highly traditional characteristics of contemporary Chinese society exacerbate the impact of negative workplace gossip on the emotional exhaustion of the gossip target. On the other hand, leaders in Chinese organizations cherish their reputations and are expected to be sage-like (Fu et al., 2010). Although supervisors have higher organizational status and formal power than subordinates, Chinese employees emphasize guanxi and mianzi in the interaction between supervisors and subordinates (Buckley et al., 2006). Because the spread of negative workplace gossip is relatively hidden (Foster, 2004; Foster and Rosnow, 2006), supervisors tend to take more subtle and covert actions to protect themselves and fight back against their subordinates. Supervisor undermining is the intentional behavior of supervisors in the workplace to prevent subordinates from establishing and maintaining positive interpersonal relationships, thereby preventing subordinates from achieving job success and a good reputation (Duffy et al., 2002; Frazier and Bowler, 2015). In view of the characteristics of undermining, the degree of harm is generally slight and usually involves a certain degree of concealment, but the effect is persistent and is not easy to detect (Duffy et al., 2002); supervisors are likely to engage in undermining to retaliate against subordinates who spread negative workplace gossip and to further maintain their self-esteem and authority (Baumeister et al., 2004). To avoid a further loss of valuable resources, undermining should be taken to protect and restore resources in stressful and injustice situations (Halbesleben et al., 2014). In addition, supervisors may strike back at subordinates by hiding the information employees need to do their jobs and reducing resources support for employees (Burris, 2012).

Although perceived subordinates’ negative workplace gossip can trigger negative emotions and experiences in supervisors and lead to negative behaviors of supervisors toward subordinates, its effect is also affected by some factors. Mindfulness describes an individual’s trait as “uncritical acceptance of current events and experiences without evaluation, judgment, and cognitive filtering” (Glomb et al., 2011). Studies have shown that trait mindfulness can help employees reduce stress (Hannes et al., 2013), burnout levels and negative emotions (Flook et al., 2013; Roche et al., 2014), and improve well-being and job performance (Dane and Brummel, 2014). Supervisors’ understandings and cognitions of subordinates’ negative workforce gossip vary with their level of mindfulness, which leads to differences in emotional exhaustion (Hülsheger et al., 2013). We infer that when a supervisor has a higher level of mindfulness, he or she is able to accept subordinates’ negative workplace gossip without judgment or evaluation, resulting in less negative emotions and a lower level of emotional exhaustion (Brady et al., 2017). The relationship between the perception of negative workplace gossip from subordinates and supervisor undermining through emotional exhaustion is weaker. Therefore, this study further explores the moderating role of supervisor mindfulness in the mediated model of subordinates’ negative workplace gossip and supervisor undermining.

Based on the above analysis, this study, from the perspective of conservation of resources theory, explores the effect and mechanism of perceived subordinates’ negative workplace gossip on supervisor undermining toward subordinates and explores the contingency role of supervisor mindfulness in it. This study aims to expand the understanding of how negative workplace gossip affects the work attitudes and behaviors of gossip targets and to provide inspiration and reference for corresponding management practices.

## Literature review and hypothesis development

### Perceived subordinates’ negative workforce gossip and supervisor undermining

According to the definition of negative gossip, subordinates’ negative gossip is perceived by their supervisors as follows: the supervisor perceives the subordinate as a gossiper who passes negative evaluations of the supervisor (the target) to other members in the organization (the recipients of gossip; Zhou et al., 2020). In fact, negative workforce gossip related to a supervisor is most likely to come from the supervisor’s direct reports (e.g., those employees who are directly abused and undermined by the supervisor; McAndrew et al., 2007; Brady et al., 2017), and supervisors’ status and power in the organization make them likely to become targets of subordinates’ workforce gossip (Naeem et al., 2019). From the perspective of social exchange theory, perceived subordinates’ negative workforce gossip, as a negative interpersonal experience (Tian et al., 2019), can easily stimulate negative reciprocity beliefs in the target and then result in them exhibiting “eye for an eye” behavior, which makes supervisors engage in destructive leadership behavior (e.g., abuse and undermining; Naeem et al., 2019; Sun et al., 2022). This is because the performance of the supervisor depends to a large extent on the work performance of the subordinates, and there is a material and emotional exchange relationship between the supervisor and the subordinates (Blau, 1964). This reciprocal relationship is reflected in that supervisors provide subordinates with work resources, opportunities, and rewards, and subordinates reward supervisors for good job performance in order to achieve organizational goals (Russell et al., 2001). If this exchange relationship is of high quality, it will also generate cognitive trust and affective trust (Johnson and Grayson, 2005; Yang et al., 2009). In the Chinese context, the role of affective trust is particularly important (Li and Liang, 2002). Once the supervisor perceives the subordinates’ negative gossip, it will greatly destroy this affective trust (Grosser et al., 2010), make the supervisor feel betrayed, cause psychological imbalance, and lead to the loss of psychological resources. At this time, the supervisor is no longer willing to provide the necessary work resources, information, and knowledge to help employees complete their work tasks. In other words, the supervisor may retaliate against the subordinate by reducing or not providing work-related resource support.

Supervisor undermining involves supervisors deliberately hindering subordinates from establishing and maintaining positive interpersonal relationships in the workplace, hindering the completion of subordinates’ work tasks, and having a negative impact on subordinates’ reputations (Duffy et al., 2002). Supervisor undermining can exclude and degrade subordinates, or they will deliberately hide information and knowledge, and reduce the provision of resources (Burris, 2012). Undermining usually has a certain degree of concealment, causes minor but persistent harm, and is not easy to detect (Duffy et al., 2002). Undermining can be used to take revenge on subordinates who spread negative workplace gossip. In addition, relationship conflict is also one of the antecedents of supervisor undermining (Eissa and Wyland, 2015). Negative workforce gossip, as a negative interpersonal experience, plays an important role in promoting supervisor undermining. In this study, the behavior of subordinates spreading negative gossip about a supervisor threatens the identity and organizational status of the supervisor, and the supervisor has a strong motivation to fight the gossip to maintain his or her own identity and authority (Felson and Steadman, 1983; Baumeister et al., 2004). In addition, due to the power gap between supervisors and subordinates, undermining imposed by supervisors on subordinates can better demonstrate power, establish authority, or restore reputation (Fatima et al., 2020), thereby preserving useful work resources and psychological resources. Therefore, supervisors may consolidate their authority and reduce the sense of threat posed by the decline in their organizational status through undermining. Based on the above analysis, we propose the following hypothesis:

*Hypothesis 1 (H1)*: Perceived subordinates’ negative workforce gossip is positively correlated with supervisor undermining.

### The mediating role of emotional exhaustion

Emotional exhaustion refers to the fatigue state resulted from employees’ excessive consumption of psychological and emotional resources, it is a stress response triggered by job pressure (Maslach and Jackson, 1981; Lam et al., 2010; McDowell et al., 2019). Studies have shown that various pressures in the workplace are important antecedents of emotional exhaustion (Cole et al., 2012). First, be gossiped about puts pressure on employees, in part due to the evaluative nature of workplace gossip (Tan et al., 2021). Negative workplace gossip typically consists of value-laden information, which results in an implicit or explicit negative evaluation of the gossip target’s behavior or reputation (Foster, 2004; Brady et al., 2017). Existing studies have also shown that perceived negative workplace gossip has a positive impact on the emotional exhaustion of the target (Wu et al., 2015; Zhou et al., 2019). Wu et al. (2015) also pointed out that in highly traditional situations (such as mainland China and Hong Kong), gossip targets are more norm-compliant and eager to respect and devote more resources to dealing with gossip, and so their emotional exhaustion is higher.

According to conservation of resources theory, exposure to negative workplace gossip puts supervisors under enormous pressure, and this negative interpersonal experience drains their emotional resources (Ellwardt et al., 2012a,b). First, when supervisors learn that they have become the target of negative workforce gossip, they have negative emotions, such as shock, anger, disappointment, and exhaustion (Naeem et al., 2019) and invest substantial emotional resources in controlling their emotions and maintaining emotional stability to deal with the negative situation. Second, supervisors may spend considerable time, energy, and other resources to identify the gossiper, the motivation behind the gossip, and the recipients of the gossip and to defend themselves and maintain their reputations, which can lead to a loss of substantial personal resources and emotional exhaustion (Wu et al., 2015).

Based on conservation of resources theory, when individuals encounter negative behaviors in the workplace and consume substantial personal resources, they usually engage in negative organizational behaviors to avoid further resource loss (Hobfoll and Walfisch, 1984). When supervisors are the subject of subordinates’ negative workforce gossip and their psychological resources are consumed, they tend to maintain their remaining resources and try to develop effective resource investment strategies to protect these resources (Hobfoll, 1989). If the interaction between supervisors and subordinates is focused on relationship harmony (Buckley et al., 2006), supervisors are likely to implement relatively covert undermining to cope with the effects of emotional exhaustion and to preserve and obtain their own work, psychological, interpersonal, and other resources (Foster, 2004; Foster and Rosnow, 2006). The main reasons are as follows. First, supervisor undermining is a destructive workplace behavior that effectively results in the gossiper being attacked, damages the gossiper’s body and mind, and reduces the gossiper’s self-efficacy (Duffy et al., 2002), thus having a compensatory effect on the supervisor and decreasing the supervisor’s emotional exhaustion level to a certain extent. Second, supervisor undermining can reveal the supervisor’s attitude to subordinates, thereby reducing the production or spreading of negative workforce gossip and may also increase the gossiper’s turnover intention (Greenbaum et al., 2012b), thus reducing the risk of supervisor emotional exhaustion. Supervisors can also further build reputation and authority from supervisor undermining to obtain favorable work and interpersonal resources. Based on the above analysis, we propose the following hypothesis:

*Hypothesis 2 (H2)*: Supervisor emotional exhaustion mediates the relationship between perceived subordinates’ negative workforce gossip and supervisor undermining.

### The moderating effect of supervisor mindfulness

Mindfulness, as a trait, is defined as “a purposeful focus on an awareness of the present moment and a curious and non-judgmental acceptance of present experience” (Glomb et al., 2011). Trait mindfulness is a relatively stable personality disposition (Brown et al., 2007; Dane, 2011), and mindfulness is a valuable concept for understanding relatively stable differences in people’s attitudes, feelings, and behaviors (Chiesa and Serretti, 2009). According to conservation of resources theory, this kind of awareness of conscious attention to the moment and acceptance without evaluation can help supervisors maintain stable psychological capital and improve their well-being (Brady et al., 2017). In addition, studies have also shown that mindfulness can effectively increase resilience (Malinowski and Lim, 2015), reduce negative mental state (e.g., stress, burnout levels, and negative emotions; Brady et al., 2017; Greengard, 2001; Ruocco and Direkoglu, 2013), and effectively control negative reactions to deviant behaviors.

Individuals with higher levels of mindfulness tend to pay closer attention to their experiences and events around them, avoid possible negative reactions (Brown and Ryan, 2003; Good et al., 2016) and are more able to deal with possible negative effects (Brady et al., 2017). Moreover, when supervisors with a high level of mindfulness perceive subordinates’ negative workplace gossip, they take it as a fact, accept it without judgment; without analysis; and become more focused on the moment, event, and task (Weick and Putnam, 2006). Accordingly, they will have fewer negative emotions (Long and Christian, 2015) and relatively low stress, with correspondingly lower levels of emotional exhaustion. In contrast, supervisors with low levels of mindfulness do not pay attention to the present moment and focus on negative evaluation information in negative workplace gossip (Good et al., 2016), which results in a higher degree of loss of psychological resources and emotional exhaustion and is more likely to target their subordinates for revenge. Therefore, trait mindfulness can help supervisors effectively deal with the pressure of negative workplace gossip and reduce the negative effect of this gossip consuming personal resources.

*Hypothesis 3 (H3)*: Supervisor mindfulness moderates the positive relationship between perceived subordinates’ negative workforce gossip and supervisor emotional exhaustion; that is, the higher the level of supervisor mindfulness is, the weaker the positive relationship between perceived subordinates’ negative workforce gossip and supervisor emotional exhaustion. Conversely, when the supervisor’s level of mindfulness is low, the stronger the positive relationship between perceived subordinates’ negative workforce gossip and supervisor emotional exhaustion.

Based on the relationship proposed by Hypothesis 2 and Hypothesis 3, this study predicts that supervisor mindfulness moderates the mediation of supervisor emotional exhaustion between perceived subordinates’ negative workforce gossip and supervisor undermining, which constitutes a moderated mediation model.

When the gossip target (supervisor) perceives negative workplace gossip, the supervisor experiences psychological tension, negative emotions (Naeem et al., 2019), reputation damage, ego depletion (Cheng et al., 2022), reduced organizational identification (Yao et al., 2020), a large loss of personal resources, and emotional exhaustion. Based on conservation of resources theory, supervisors tend to engage in covert undermining behaviors to deal with the emotional exhaustion caused by subordinates spreading their negative gossip, maintain their reputation, prevent the spread of work gossip, and maintain psychological balance to persevere and gain work, psychology, interpersonal, and other valuable resources. As mentioned above, trait mindfulness is a mindful disposition, a conscious awareness of the present moment and acceptance without evaluation that helps supervisors maintain stable psychological capital (Good et al., 2016). Supervisors with a higher level of mindfulness have more adequate psychological resources, accept workplace gossip without evaluation, and pay closer attention to current events and experiences. It helps employees establish a greater buffer space between negative emotions and their own reactions (Liang et al., 2017), reduce impulsive cognitions or negative behaviors, and improve self-adjustment and management functions, thereby weakening the relationship between negative workplace gossip and emotional exhaustion. In addition, supervisors with high mindfulness generally have higher levels of empathy and compassion (Dekeyser et al., 2008) and engage in more behaviors that help build relationships (Mahsud et al., 2010). More importantly, supervisors with high mindfulness are able to deal with negative events at work and avoid retaliation (Long and Christian, 2015). In contrast, when the level of supervisory trait mindfulness is low, the supervisor lacks sufficient attention to any work affairs and interpersonal interactions at the moment, is easily affected by negative events such as negative workplace gossip, and has a high degree of emotional exhaustion. Because supervisors with low mindfulness lack empathy and loving kindness (Dekeyser et al., 2008), they are also less likely to initiate interpersonal behaviors (Mahsud et al., 2010). The effect of indirect influence on supervisor undermining is also weakened accordingly. We further propose another hypothesis:

*Hypothesis 4 (H4)*: Supervisor mindfulness moderates the mediating effect of supervisor emotional exhaustion on perceived subordinates’ negative workforce gossip and supervisor undermining; that is, the higher the supervisor mindfulness is, the weaker the mediating effect; the lower the supervisor mindfulness is, the stronger the mediating effect.

**Figure 1:**
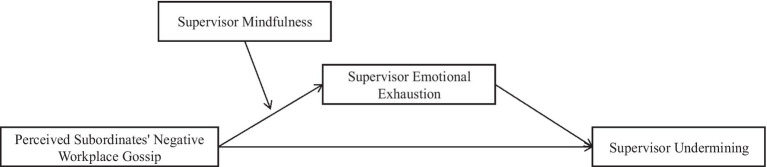
The research conceptual model.

We speculate that perceived negative workplace gossip from subordinates will increase supervisors’ emotional exhaustion to a certain extent and lead to supervisors’ repressive behavior toward subordinates. Supervisor mindfulness moderates the positive relationship between perceived subordinates’ negative workforce gossip and supervisor emotional exhaustion, Supervisor mindfulness moderates the mediating effect of supervisor emotional exhaustion on perceived subordinates’ negative workforce gossip and supervisor undermining. Altogether, we summarize our research variables and hypotheses in a conceptual framework in Figure 1.

## Materials and methods

### Sample and design

Given the sensitivity of this research topic, we had our research proposal approved by the Academic Committees of our school before collecting data. The survey respondents were mainly from 35 manufacturing enterprises in Jiangsu and Anhui Provinces in China, and the survey respondents were mainly managers at all levels and their direct subordinates. This is because, as a labor-intensive industry, employees in the manufacturing industry have a high degree of interdependence of tasks (Kiggundu, 1983) and a high degree of clustering in the workplace, providing a breeding ground for the spread of workplace gossip. To avoid the influence of common method bias on the research results as much as possible, the supervisor and his or her subordinate were paired into groups, and the independent variables and dependent variables were measured separately by the supervisors and their direct subordinates. This study adopted a double-blind approach to data acquisition, with subordinates and supervisors reporting anonymously and matched *via* employee numbers. In addition, with the cooperation of the company’s human resource department, we carried out three consecutive sessions of psychological counseling activities, openly discussing workplace gossip, asking employees to fill out questionnaires at the end of the activity, and allowing employees to obtain two chocolates when they leave the meeting room.

A total of three questionnaire surveys were conducted: 550 questionnaires were distributed in the first survey and were mainly used to investigate the supervisors’ demographic information (gender, age, education, and organizational tenure), perceived subordinates’ negative workforce gossip, and supervisor mindfulness, and 474 valid questionnaires were obtained (response rate 86.18%). The second survey was conducted after a 2-week interval, and 474 questionnaires were distributed based on the valid questionnaires returned in the first survey and were mainly used to investigate the emotional exhaustion of the supervisors. Afterward, 406 valid questionnaires were obtained (response rate 85.65%). The third survey was conducted at 2-week intervals based on the second survey, and a total of 406 questionnaires were completed by a direct subordinate of the supervisor. It was mainly used to investigate the supervisor undermining perceived by subordinates. After sorting, 362 valid questionnaires were obtained (response rate 89.16%). In the sample, there were 235 males (64.9%) and 127 females (35.1%). The average age of supervisors was 33.99 years, and the SD was 8.12; 74.8% supervisors had a bachelor’s degree or higher, the supervisors’ average organizational tenure was 6.77 years, and the SD was 8.37.

### Measures

To ensure the reliability and validity of the measurements, we adopted well-established scales developed and used by previous researchers. All scale items underwent a translation/back-translation procedure to translate English language measures into the Chinese language (Brislin, 1986) using competent bilingual individuals. Back-translation was conducted, and one Chinese associate professor compared the original and back-translated versions. At the same time, we referred to relevant scales in Chinese authoritative journals to ensure the applicability of foreign scales in the Chinese context. We used a Likert seven-point scale with 7 for “strongly agree” and 1 for “strongly disagree,” except for demographic items.

Perceived negative workplace gossip was assessed using a scale adapted from the three-item scale of Chandra and Robinson (2009) and applied by Wu et al. (2015) in a workplace context in China. A sample item is “In the past 6 months, subordinates communicated damaging information about me in the workplace” (*α* = 0.790; Nunnally, 1978).

Emotional exhaustion was adopted from the scale developed by Watkins et al. (2014), which contains three items, such as “I feel emotionally drained from my work” (*α* = 0.844).

Undermining was used in the scale developed by Vinokur and Ryn (1993), which contains five items, such as “In the workplace, my supervisor acts in an unpleasant or angry manner toward me” (α = 0.792).

Mindfulness was measured by the scale developed by Brown and Ryan (2003), referring to the study of Schuh et al. (2017), which used five items such as “I rush through activities without being truly attentive to them” (α = 0.776).

#### Control variables

This study selected the supervisor’s gender, age, education, and organizational tenure as control variables to obtain more accurate results. Studies have shown that female managers are more vulnerable to negative workplace gossip due to traditional cultural prejudice against gender (Michelson and Mouly, 2000). And the female employees are more likely to be influenced by negative events (Warren and Catherine, 1999). The employee demographic variables, such as gender, age, education, and organizational tenure are important factors influencing emotional exhaustion (Maslach and Jackson, 1982; Maslach et al., 2001; Houkes et al., 2003).

## Results

### Preliminary analyses

Table 1 demonstrates the correlation coefficients, means, and SDs of all variables. As shown in Table 1, perceived subordinates’ negative workplace gossip and supervisor emotional exhaustion have a significantly positive correlation (γ = 0.259, *p* < 0.01), perceived subordinates’ negative workplace gossip and supervisor undermining are significantly positively correlated (γ = 0.313, *p* < 0.01), and supervisor emotional exhaustion and supervisor undermining have a significantly positive correlation (γ = 0.261, *p* < 0.01). These results provide preliminary support for our hypotheses of the main effect and the mediation effect.

**Table 1:** Descriptive statistical analysis and correlations (*N* = 362).

**Variable**	1	2	3	4	5	6	7	8
1. Supervisor Gender								
2. Supervisor Age	−0.081	1.000						
3. Supervisor Education	0.106^*^	−0.403^**^	1.000					
4. Supervisor Tenure	−0.016	0.688^**^	−0.247^**^	1.000				
5. PSNWG	−0.047	−0.090	−0.068	−0.060	1.000			
6. SEE	−0.093	0.121^*^	−0.326^**^	0.016	0.259^**^	1.000		
7. SM	0.031	0.242^**^	−0.138^**^	0.238^**^	−0.072	−0.005	1.000	
8. SU	0.032	−0.106^*^	0.029	−0.001	0.313^**^	0.261^**^	−0.251^**^	1.000
M	1.350	33.990	2.720	6.772	2.421	3.343	5.064	2.672
SD	0.478	8.118	0.661	8.373	1.045	1.575	1.043	1.025

PSNWG, perceived subordinates’ negative workplace gossip; SEE, supervisor emotional exhaustion; SU, supervisor undermining; and SM, supervisor mindfulness.

** *p* < 0.01;

* *p* < 0.05 (two-tailed).

Since the data in this study were all derived from self-reports by employees and supervisors, the results of the study may be affected by common method bias. Exploratory factor analysis was carried out on the survey data by SPSS 25.0 software, and the results showed that four factors were separated out. The first factor explained only 25.23% of the total difference, which did not exceed 40% (Podsakoff et al., 2003), and the common variance explained rate of the four factors was 62.43%, indicating that there was no obvious common method bias in this study.

### Reliability and validity test

This study used SPSS 25.0 software to analyze the reliability of the scale. The Cronbach’s α coefficient of perceived subordinates’ negative workplace gossip, supervisor emotional exhaustion, supervisor undermining, and supervisor mindfulness were 0.790, 0.844, 0.792, and 0.776, respectively. The reliability coefficients of the four scales were all greater than 0.7, indicating good internal consistency. Confirmatory factor analysis was performed on the four variables using Mplus7 software. The results are shown in Table 2. Compared with other models, it is obvious that the four-factor model has the best fit, and all indicators are ideal, χ^2^/df = 2.324; CFI = 0.935; TLI = 0.920; SRMR = 0.051; RMSEA = 0.061. This indicates that the four variables selected in this study have good discriminant validity and represent four different constructs.

**Table 2:** The results of confirmatory factor analysis (CFA).

Model	χ^2^	df	χ^2^/df	CFI	TLI	SRMR	RMSEA
Model 1	227.726	98	2.324	0.935	0.920	0.051	0.061
Model 2	632.860	101	6.266	0.733	0.683	0.093	0.121
Model 3	629.180	101	6.230	0.735	0.685	0.088	0.120
Model 4	888.284	103	8.624	0.606	0.541	0.107	0.145
Model 5	1336.185	104	12.848	0.382	0.286	0.144	0.181

Model 1 (four factors): perceived subordinates’ negative workplace gossip, supervisor emotional exhaustion, supervisor undermining, and supervisor mindfulness.

Model 2 (three factors): perceived subordinates’ negative workplace gossip, supervisor emotional exhaustion + supervisor undermining, and supervisor mindfulness.

Model 3 (three factors): subordinates’ negative workplace gossip + supervisor emotional exhaustion, supervisor undermining, and supervisor mindfulness.

Model 4 (two factors): perceived subordinates’ negative workplace gossip + supervisor emotional exhaustion + supervisor undermining, supervisor mindfulness.

Model 5 (one factor): perceived subordinates’ negative workplace gossip + supervisor emotional exhaustion + supervisor undermining + supervisor mindfulness.

### Main effect and mediation effect tests

The results of the regression analysis using SPSS 25.0 are shown in Table 3. First, M2 shows that subordinates’ perceived negative workplace gossip has a significant positive impact on supervisor undermining (M2, β = 0.309, *p* < 0.001); thus, Hypothesis 1 was verified.

**Table 3:** The results of hierarchical regression analysis.

Variables	SU				SEE				
	M1	M2	M3	M4	M5	M6	M7	M8	M9
Gender	0.020	0.034	0.037	0.045	−0.055	−0.044	−0.055	−0.043	−0.037
Age	−0.208^**^	−0.161^*^	−0.226^**^	−0.185^*^	0.059	0.095	0.059	0.098	0.091
Education	−0.023	0.015	0.079	0.088	−0.322^***^	−0.293^***^	−0.322^***^	−0.295 ^***^	−0.296^***^
Tenure	0.137	0.133	0.170^*^	0.160^*^	−0.105	−0.108	−0.105	−0.104	−0.103
PSNWG		0.309^***^		0.250^***^		0.239^***^		0.237^***^	0.220^***^
SEE			0.314^***^	0.249^***^					
SM								−0.026	−0.014
PNSWG*SM									−0.142^**^
*R* ^2^	0.022	0.115	0.109	0.167	0.115	0.171	0.115	0.172	0.191
Δ*R*^2^		0.093^***^	0.087^***^	0.052^***^		0.056^***^		0.057^***^	0.019^**^
*F*	2.005	9.277^***^	8.748^***^	11.822^***^	11.642^***^	14.684^***^	11.642^***^	12.257^***^	11.966 ^***^

PSNWG, perceived subordinates’ negative workplace gossip; SEE, supervisor emotional exhaustion;

SU, supervisor undermining; SM, supervisor mindfulness.

*** *p* < 0.001;

** *p* < 0.01;

*p < 0.05 (two-tailed).

This study uses the hierarchical regression method proposed by Baron and Kenny (1986) to test the mediation effect. The results of hierarchical regression are shown in Table 3. Among them, under the premise that the main effect is significant, subordinates’ negative workplace gossip has a significant positive relationship with supervisor emotional exhaustion (M6, β = 0.239, *p* < 0.001), and emotional exhaustion has a significant positive effect on supervisor undermining (M3, β = 0.314, *p* < 0.001). After adding the mediator variable of supervisor emotional exhaustion based on M2, perceived subordinates’ negative workplace gossip still had a significant effect on supervisor undermining (M4, β = 0.250, *p* < 0.001), but the significance was reduced (M2, β = 0.309, *p* < 0.001). The results suggest that supervisor emotional exhaustion partially mediates the relationship between perceived subordinates’ negative workplace gossip and supervisor undermining. Hypothesis 2 was supported.

In addition, this study used bootstrapping to analyze the significance of the indirect effects (Table 4). The 95% CI [0.027, 0.098] did not contain 0; thus, the indirect effects are significant. Furthermore, the value of the indirect effects is 0.058. After controlling for the mediation variable, the direct effect of perceived subordinates’ negative workplace gossip on supervisor undermining through supervisor emotional exhaustion was significant, and the 95% CI [0.147, 0.343] did not contain 0, indicating that the mediation role is partial. Therefore, Hypothesis 2 was supported.

**Table 4:** The results of the mediation effect analysis.

	Path	Effect	Standard error	95% CI	
Indirect effect	PSNWG→SEE→SU	0.245	0.050	0.147	0.343
Direct effect	PSNWG→SU	0.058	0.018	0.027	0.098

PSNWG, perceived subordinates’ negative workplace gossip; SEE, supervisor emotional exhaustion, SU, supervisor undermining; and SM, supervisor mindfulness.

**Figure 2:**
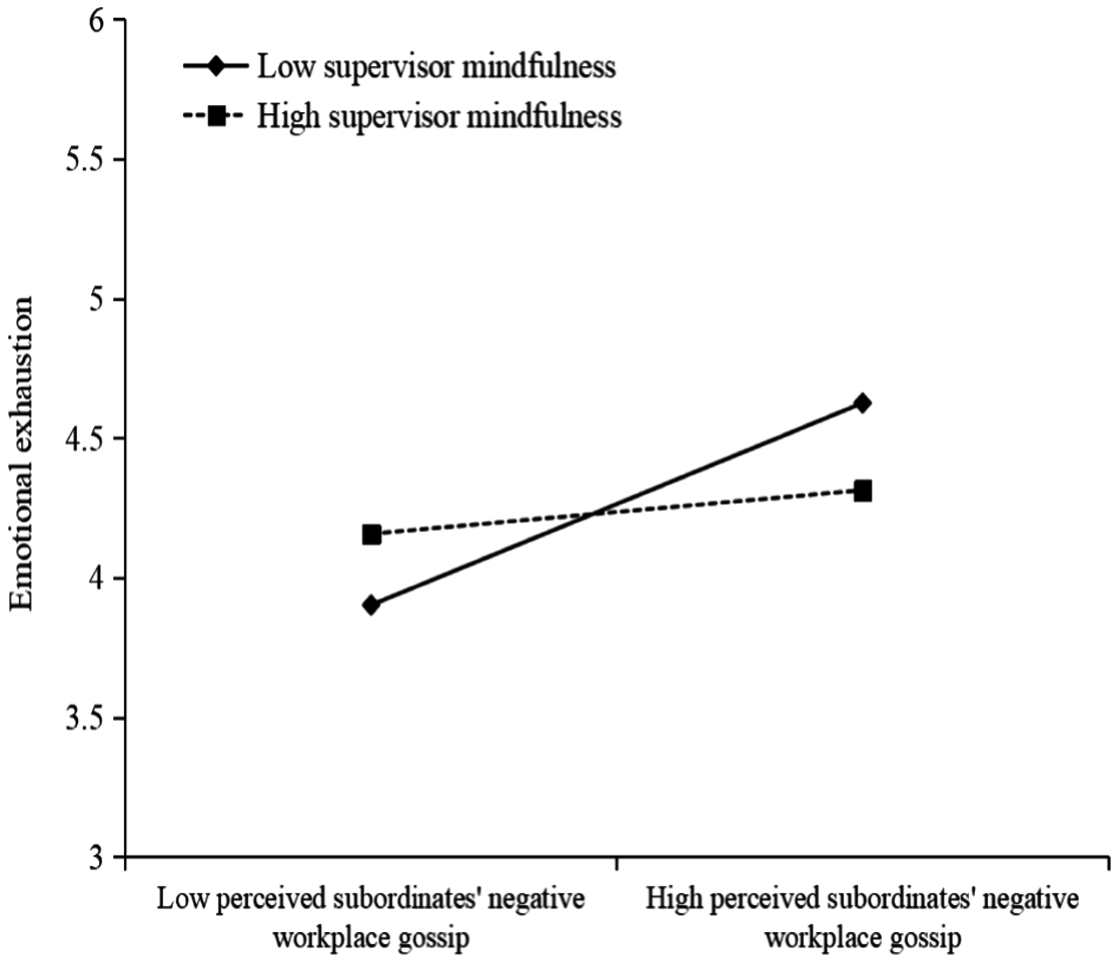
The moderating effect of supervisor mindfulness.

Third, a moderating effect test was conducted. Model 9 (Table 3) shows that the regression coefficient of perceived subordinates’ negative workplace gossip*supervisor mindfulness is significant (β = −0.142, *p* < 0.01), which indicates that supervisor mindfulness significantly moderates the relationship between perceived subordinates’ negative gossip and supervisor emotional exhaustion. Hypothesis 3 was thus verified. To more intuitively show the moderating effect of supervisor mindfulness on perceived subordinates’ negative workplace gossip and supervisor emotional exhaustion, this paper draws a diagram of the moderating effect (see Figure 2) and performs a simple slope test. Figure 2 shows that when supervisors have a higher level of mindfulness, the impact of perceived subordinates’ negative workplace gossip on supervisors’ emotional exhaustion is weaker. Furthermore, the greater the slope of the straight line is, the higher the level of mindfulness.

To test Hypothesis 4, this study tested the moderated mediation effect. The results of the bootstrapping of conditional indirect effects are shown in Table 5. Several specific values of the moderator variables were selected to represent the interaction at higher, average, and lower levels, respectively, with one standard deviation above the mean, equal to the mean, and one SD below the mean as the criterion (Baron and Kenny,1986).When the level of supervisor mindfulness is low, the indirect effect of supervisor emotional exhaustion between subordinates’ perceived negative workplace gossip and supervisor undermining is significant (*b* = 0.077, 95% CI [0.040, 0.127] does not contain 0); when the level of supervisor mindfulness is higher, the mediating effect of supervisor emotional exhaustion between subordinates’ perceived negative workplace gossip and supervisor undermining is not significant (*b* = 0.023, 95% confidence interval [−0.008, 0.065] contains 0). Thus, Hypothesis 4 was partially validated.

**Table 5:** Results of the moderated mediation effect analysis.

Moderators	Effect value	Standard error	95% CI	
Low supervisor mindfulness	0.077	0.022	0.040	0.127
Middle supervisor mindfulness	0.055	0.017	0.027	0.095
High supervisor mindfulness	0.023	0.018	−0.008	0.065

## Discussion

As supervisors become the target of potential subordinates’ negative workplace gossip, it is important to understand the impact of subordinates’ negative workplace gossip on supervisors’ work-related attitudes and behaviors and to find ways to reduce its adverse effects. Drawing on conservation of resources theory, this study provides insights into how this experience causes emotional exhaustion and promotes undermining. In addition, by investigating the moderating roles of supervisor mindfulness, we have enhanced our understanding of the boundary conditions of the process. Since negative workplace gossip places supervisors in an unfavorable position and causes them to be exhausted, leaders need to take this phenomenon seriously and make efforts to address its negative effects. This study enriches the literature and suggests future research directions.

### Theoretical implications

The theoretical contributions are mainly manifested in the following: First, this study expands our understanding of the negative consequences of negative workplace gossip from the perspective of the target, with particular attention to the attitudes and behavioral responses of supervisors as the target of negative workplace gossip. As mentioned above, the components of negative workplace gossip include gossipers, gossip targets, and gossip receivers (Dores Cruz et al., 2021). Many studies have revealed the motivations of gossipers, such as gaining power and information and developing interpersonal relationships (Tassiello et al., 2018); however, gossipers reduce trust in the organization due to cognitive dissonance and thus are reluctant to share knowledge (Zou et al., 2020). The gossip receiver may cause secondary damage to the gossip target, such as withholding target support, engaging in target exclusion, or targeting negative gossip (Zhou et al., 2020; Guo et al., 2021). From the perspective of negative workplace gossip targets, previous studies have mostly studied employees as the target of gossip and have confirmed the detrimental impact of gossip on targets’ attitudes and behaviors, such as reducing their organization-based self-esteem (Zhou et al., 2019); lowering organizational identification (Ye et al., 2019); promoting negative mood (Babalola et al., 2019); and causing a decline in innovative behavior (Zhou et al., 2019), proactive behavior (Wu et al., 2015; Tian et al., 2019), and organizational citizenship behavior (Wu et al., 2016; Kong, 2018; Ye et al., 2019). This study turns the attention to negative workplace gossip behaviors from the perspective of subordinate-supervisor relationships, broadening the research perspective and addressing gaps in the research. Given that supervisors are one of the main targets of negative workplace gossip (Brady et al., 2017), the study investigated supervisors’ work behaviors by taking negative workplace gossip spread by subordinates as a distal influencer and supervisors’ emotional exhaustion as a proximal influencer. Our findings respond to organizational realities and advance research on the perspective of supervisors as targets of negative workplace gossip (Naeem et al., 2019).

Second, this study extends the research on the antecedent variables of supervisor undermining. Studies have shown that supervisor inhibition negatively affects employees’ self-esteem, creativity, and overall job performance (Eissa et al., 2017) and induces employee obedience behavior (Fatima et al., 2020). A review of previous studies on undermining behavior reveals that, compared with the abundance of outcome variables, research on the antecedent variables responsible for undermining appears to be very scarce. Therefore, in recent years, some scholars have called for additional exploration of its antecedent mechanism (Duffy et al., 2006, 2012). Existing research mostly discusses the internal factors of supervisors (e.g., bottom-line mentality, core self-evaluations, and conscientiousness; Greenbaum et al., 2012a) affecting undermining, but little is known about how subordinates’ related behaviors in the workplace induces supervisor undermining. From the perspective of subordinate behavior, this study explores the predictive effect of perceived negative workplace gossip on supervisor undermining. Thus, our findings advance current research on perceived subordinates’ negative gossip as a distal antecedent, and we also improve our understanding of the underlying mechanism of supervisor emotional exhaustion as a proximal antecedent of supervisor undermining.

Third, as an effective moderator, supervisor mindfulness enriches the theoretical study of moderator variables of negative workplace gossip. Negative gossip in the workplace exists objectively and cannot be completely eliminated (Noon and Delbridge, 1993). Many studies have reduced the negative effects of negative workplace gossip and have further explored the boundary effects of personality traits (such as emotional regulation, extroversion, traditionality, self-monitoring, and impression management tactics; Wu et al., 2015; Liu et al., 2019; Naeem et al., 2019; Xie et al., 2019a). This study shows that supervisor mindfulness plays a role between subordinates’ negative workplace gossip and supervisor undermining. Specifically, when a supervisor’s level of mindfulness is high, the supervisor pays more attention to the work at hand and what is happening at the moment and does not comment on or respond to the negative workplace gossip of subordinates; thus, the negative effect is lower, and the supervisors themselves also have less negative impact. This study verifies and supports the accuracy of the above results by incorporating perceived subordinates’ negative workplace gossip, supervisor emotional exhaustion, and supervisor mindfulness into the same framework and expands the study of the boundary conditions of perceived subordinates’ negative workplace gossip and supervisor undermining.

### Practical implications

Negative gossip in the workplace exists objectively and cannot be completely eliminated (Noon and Delbridge, 1993). Our research findings carry important implications for organization practitioners. In management practice, both negative workplace gossip and supervisor undermining are considered negative workplace behaviors, which can have a serious negative impact on the organizational environment and work efficiency (Chandra and Robinson, 2009; Fatima et al., 2020), so appropriate measures need to be taken to cope with these negative impacts (Xie et al., 2019a). Since our research samples are from labor-intensive enterprises in China, work tasks are highly interdependent, employees collaborate in teams, and employees communicate closely. Employees participate in negative workplace gossip based on friendship ties, transmit information, and generate affective trust (Waddington, 2005; Grosser et al., 2010). The results of this study have practical implications for labor-intensive enterprises that value harmonious relationships (Wu et al., 2015).

First, establish effective communication channels, create an inclusive cultural atmosphere, and actively divert negative gossip in the workplace. Organizations should establish effective communication channels, create unimpeded formal communication channels and informal communication networks, solve problems such as information asymmetry, and reduce the breeding grounds for negative workplace gossip (Foster, 2004). Moreover, organizations should actively create an inclusive culture, provide managers and employees with diversity training and coaching, and use 360-degree reviews of managers and employees to avoid negative workplace gossip. In a training program, organizations can advise employees on how negative workplace gossip can be far more impactful than they may think and advise employees to resolve issues through formal discussions. Finally, for supervisors who have long been plagued by negative workplace gossip, after investigation and research, the organization should clarify and effectively safeguard the interests of the organization and individuals in a timely manner.

Furthermore, organizational support (Xie et al., 2019b; Zhou et al., 2020) and coaching training should be provided to protect supervisors from emotional exhaustion. On the one hand, organizations should provide a variety of beneficial resources to help supervisors recover physically and mentally and reduce their emotional exhaustion, pay more attention to supervisors’ health costs, create a fair and just organizational culture and values, and support supervisors in obtaining positive resources such as material, work, psychology, and interpersonal relationships. On the other hand, organizations should actively protect and train supervisors and employees to prevent resource drain, guide supervisors to rationally view subordinates’ negative workplace gossip, encourage employees to communicate face-to-face, and effectively reduce the emotional exhaustion of supervisors.

Last, the level of mindfulness of supervisors should be improved, and mindfulness training (Glomb et al., 2011) and talent selection mechanisms should be used rationally. Organizations can provide specialized mindfulness training (Baas et al., 2014), including mindfulness meditation and mindfulness yoga, for supervisors with low levels of mindfulness to help them focus their attention, and thereby reducing the perception of negative workplace gossip. Furthermore, supervisor support can not only reduce emotional exhaustion but also foster mindfulness (Narayanan and Moynihan, 2006). In addition, when an organization promotes and selects talent, it should not only examine the performance of employees but also include the level of mindfulness in the assessment scope to obtain more high-quality supervisor resources from the source.

### Limitations and future research

First, negative workplace gossip and supervisor undermining are sensitive topics that can easily lead to resistance from research subjects. Subjects may deliberately conceal the true situation, which will lead to certain deviations in the research results, and the measurement of supervisor undermining is measured by the corresponding responses of the supervisors. Reports made by direct subordinates may deviate from the actual results. Therefore, future research can use implicit measurement and multisource evaluation to explore the behavioral relationships between supervisors and subordinates.

Second, the control variables involved in this study included only demographic variables, and emotional exhaustion may be affected by organizational characteristics (such as organizational justice and organizational support; Michàl et al., 2001) and job characteristics (such as role overload; Zapf et al., 2001). Future research should consider including these factors as control variables in the research model to improve the reliability of the research.

Third, our sample is from manufacturing enterprises in China, which provides an ideal setting for the study of the negative workplace gossip. However, differences in industry characteristics, cultural differences between China and Western countries may raise concerns of generalizability. For example, some studies have focused on front-line service employees in the service industry to explore the impact of negative workplace gossip on service performance (Tian et al., 2019; Ye et al., 2019). Furthermore, Chinese people uphold traditional values and follow social norms, and the negative effects of negative workplace gossip are more significant (Wu et al., 2015; Liu et al., 2019). In Western countries, where individualism is higher, interdependence and affiliation appear less important (Hofstede et al., 2010), Western people are more independent and therefore more likely to ignore other people’s gossip about them (Lee and Tiedens, 2001; Wu et al., 2012). Hence, we should investigate in a wider range of industries and jobs, and a cross-cultural research design is required to examine the generalizability of our model.

## Conclusion

Drawing on conservation of resources theory, we examined the influencing mechanism and conditions of perceived subordinates’ negative workplace gossip on supervisor undermining. With 362 valid data samples, we found that (1) perceived subordinates’ negative gossip has a significant positive effect on supervisor undermining; (2) perceived subordinates’ negative workplace gossip predicts supervisor undermining through supervisor emotional exhaustion; and (3) supervisor mindfulness buffers the positive relationship between perceived subordinates’ negative workplace gossip and supervisor undermining.

These findings provide important implications to the literature on negative gossip in the workforce. They also contribute to the growing body of knowledge on how subordinates’ negative workplace gossip leads to supervisor undermining. Meanwhile, we discovered that supervisor mindfulness, as a trait, can reduce the negative impact of negative workplace gossip from subordinates. The above conclusions also provide inspiration for individuals and organizations to deal with negative workplace gossip, purify the organizational climate, and improve individual well-being.

## Data availability statement

The original contributions presented in the study are included in the article/supplementary material, further inquiries can be directed to the corresponding authors.

## Ethics statement

The studies involving human participants were reviewed and approved by Ethics Committee of the School of Economics and Management in Jiangxi Science and Technology Normal University. The patients/participants provided their written informed consent to participate in this study.

## Author contributions

HZ and LZ: conceptualization and writing—review and editing. LZ: methodology. HZ and JL: resources, writing—original draft preparation, and funding acquisition. HZ, LZ, and JL: investigation. All authors contributed to the article and approved the submitted version.

## Funding

This study was supported by National Natural Science Foundation of China (72262020, 72262010, 71972139 and 71862019).

## Conflict of interest

The authors declare that the research was conducted in the absence of any commercial or financial relationships that could be construed as a potential conflict of interest.

## Publisher’s note

All claims expressed in this article are solely those of the authors and do not necessarily represent those of their affiliated organizations, or those of the publisher, the editors and the reviewers. Any product that may be evaluated in this article, or claim that may be made by its manufacturer, is not guaranteed or endorsed by the publisher.

## Appendix

Negative workplace gossip

① In the past six months, subordinates spread unfavorable gossip about me in the workplace.
② In the past six months, subordinates made negative allegations about me in the workplace.
③ In the past six months, subordinates communicated damaging information about me in the workplace.

Emotional exhaustion

① I feel emotionally drained from my work.
② I feel burned out from my work.
③ I feel exhausted when I think about having to face another day on the job.

Undermining

① In the workplace, my supervisor acts in an unpleasant or angry manner toward me.
② In the workplace, my supervisor makes my life difficult.
③ In the workplace, my supervisor shows he or she dislikes me.
④ In the workplace, my supervisor makes me feel unwanted.
⑤ In the workplace, my supervisor criticizes me.

Mindfulness

① I rush through activities without being really attentive to them.
② It seems that I’m “running on automatic” without much awareness of what I’m doing.
③ I do jobs or tasks automatically, without being aware of what I’m doing.
④ I drive places on “automatic pilot” and then wonder why I went there.
⑤ I find myself doing things without paying attention.
